# Transcriptome dataset of human corneal endothelium based on ribosomal RNA-depleted RNA-Seq data

**DOI:** 10.1038/s41597-020-00754-1

**Published:** 2020-11-20

**Authors:** Yuichi Tokuda, Naoki Okumura, Yuya Komori, Naoya Hanada, Kei Tashiro, Noriko Koizumi, Masakazu Nakano

**Affiliations:** 1 Department of Genomic Medical Sciences, Kyoto Prefectural University of Medicine, Kyoto, 602-8566 Japan; 2 Department of Biomedical Engineering, Faculty of Life and Medical Sciences, Doshisha University, Kyotanabe, 610-0321 Japan

**Keywords:** Transcriptomics, Gene expression, RNA sequencing, Visual system

## Abstract

The corneal endothelium maintains corneal transparency; consequently, damage to this endothelium by a number of pathological conditions results in severe vision loss. Publicly available expression databases of human tissues are useful for investigating the pathogenesis of diseases and for developing new therapeutic modalities; however, databases for ocular tissues, and especially the corneal endothelium, are poor. Here, we have generated a transcriptome dataset from the ribosomal RNA-depleted total RNA from the corneal endothelium of eyes from seven Caucasians without ocular diseases. The results of principal component analysis and correlation coefficients (ranged from 0.87 to 0.96) suggested high homogeneity of our RNA-Seq dataset among the samples, as well as sufficient amount and quality. The expression profile of tissue-specific marker genes indicated only limited, if any, contamination by other layers of the cornea, while the Smirnov-Grubbs test confirmed the absence of outlier samples. The dataset presented here should be useful for investigating the function/dysfunction of the cornea, as well as for extended transcriptome analyses integrated with expression data for non-coding RNAs.

## Abstract (summary)

**Table:** 

Measurement(s)	RNA
Technology Type(s)	RNA sequencing
Factor Type(s)	sex
Sample Characteristic - Organism	Homo sapiens

Machine-accessible metadata file describing the reported data: 10.6084/m9.figshare.13186868

## Background & Summary

The cornea is a transparent tissue located at the outermost surface of the eyeball, where it refracts light as a lens. It is comprised of five different layers: the epithelium, Bowman’s layer, stroma, Descemet’s membrane, and endothelium^1^. The corneal endothelium maintains corneal transparency by regulating the inflow and outflow of the aqueous humor to the stroma via a pump-and-barrier function. The cells of the corneal endothelium have limited proliferative capacity^2,3^; therefore, damage to the corneal endothelium by several pathological conditions, such as Fuchs endothelial corneal dystrophy (FECD), invasive cataract surgery, glaucoma surgery, and endotheliitis, can induce a loss of corneal transparency and result in severe visual disturbance^4^. The only therapeutic remedy has been transplantation of a donor cornea, and more than 50% of the indications for corneal transplantation involve corneal endothelial decompensation^5,6^.

Cell therapy for treating corneal endothelial decompensation has been investigated by many research groups^7^. Indeed, a clinical trial involving injection of cultured corneal endothelial cells into the anterior chamber of the eye has been initiated in Japan^8^. The induction of corneal endothelial cells from human pluripotent stem cells (iPSCs) has been reported, and the transplantation of those cells has been proposed as a potential decompensation therapy^9^. Along this line, many researchers have devoted their efforts to determining selective markers for the corneal endothelium to validate cultured cells for clinical use^10–15^. In addition, a candidate causative gene for FECD has been identified^16,17^ that affects ~4% of the population over the age of 40 in the United States^18–20^ and is responsible for most common corneal dystrophies. This identification of a causative gene, in turn, has led to the elucidation of the pathophysiology and to the proposal of multiple potential therapeutic modalities^21–23^. In line with this recent accelerating research in the field of corneal endothelial decompensation, the establishment of a rigid gene expression database is anticipated.

Gene expression data derived from multiple species and tissues are useful for revealing the molecular pathogenesis of diseases and are now increasingly available from public databases. However, gene expression data for human ocular tissues in particular, such as corneal endothelial cells, remains limited. For example, no ocular data are listed on the GTEx Portal (https://www.gtexportal.org/), while only summarized data derived from whole eye, retina, and some ocular cell lines are listed on the ENCODE project (https://www.encodeproject.org/). In terms of the corneal endothelium, two records are found in the “Publication” of “Data Type” deposited in ENCODE, and one article describes mRNA expression^24^ by referring to other published RNA-Seq data^25^. On the other hand, there are some published articles describing the results of RNA-Seq data derived from corneal endothelium (Supplementary Table 1), although all of the data were based on the sequencing libraries generated from poly-A selected mRNA and sequenced by single-end 50-bp reads^25–29^. In addition, some of the data were produced from cell lines or after performing *ex vivo*/primary culture, which could induce artificial gene expression. Recently, RNA-Seq analysis of the human ocular tissues, including cornea, has been reported as a result of pair-end 150-bp sequencing, although they used the whole cornea tissues without separating into the different layers^30^. Consequently, our RNA-Seq dataset based on the pair-end 100-bp sequencing using the ribosomal RNA-depleted total RNA should be useful for analyzing unbiased expression of not only coding genes but also non-coding RNAs of human corneal endothelial cells.

Therefore, in this data descriptor, our goal was to provide a transcriptome dataset based on ribosomal RNA-depleted RNA-Seq data derived from the corneal endothelium of seven normal Caucasian donors to serve as a gold standard for expression analyses. To this end, we paid strict attention to the selection criteria of the human donors (i.e., a narrow range of age distribution and an almost equal number of each gender), as well as to the qualities of the extracted total RNA and the generated RNA-Seq data, through several quality-control (QC) procedures. Consequently, the dataset established here should be a useful, reliable, and robust tool for understanding the cellular characteristics of the cornea endothelium, as well as a reference control for revealing the molecular mechanisms of disease pathogenesis in corneal dystrophies.

## Methods

### Ethics statement

The human tissue used in this study was handled in accordance with the tenets set forth in the Declaration of Helsinki. Informed written consent to utilize donor corneal tissue for eye research was obtained from the next of kin of all deceased donors. All corneal tissue was recovered under the tenets of the Uniform Anatomical Gift Act (UAGA) of the particular state in which the donor consent was obtained and the tissue was recovered.

### Corneal endothelial tissues

Normal human donor corneas were obtained from CorneaGen^TM^ (https://corneagen.com/, Seattle, WA). All corneas had been stored at 4 °C in storage medium (Optisol-GS; Bausch & Lomb, Rochester, US-NY) for less than 14 days before use for experiments. Corneas derived from 7 donors (3 males and 4 females of Caucasian descent; age range: 48–69 years old) were used in the study. Descemet’s membrane, including the corneal endothelium, was stripped from the donor corneas. The corneal endothelium was then lysed in 700 μL of QIAzol lysis reagent (Qiagen, Valencia, CA), homogenized with a vortex mixer for 30 seconds, and stored at −80 °C until used for experiments.

### Total RNA preparation

Total RNA was extracted from each corneal endothelium with an RNeasy Mini Kit (Qiagen). Briefly, the lysed corneal endothelium in QIAzol lysis reagent was thawed at 37 °C and incubated for 5 minutes at room temperature. Chloroform (140 μL) was added and the samples were mixed thoroughly, followed by centrifugation at 12,000 × g for 15 minutes at 4 °C. The upper layer was collected, mixed with an equal volume of 70% ethanol, and concentrated using spin columns. The final concentration of total RNA was measured with an Agilent 2100 Bioanalyzer (Agilent Technologies, Santa Clara, CA) and an RNA 6000 Pico Kit (Agilent Technologies). The quality of the total RNA was assessed by calculating the RNA integrity number (RIN) with the Agilent 2100 Expert Software (Agilent Technologies) (Table 1). The total RNA samples were snap-frozen in liquid nitrogen and stored at −80 °C until use (the mean ± SD storage period was 612.6 ± 27.3 days).

**Table 1:** Sample information.

Sample ID	Age	Sex	Total RNA		
			RIN^†^	Concentration (ng/μL)	Yield (ng)
S1	69	Female	7.6	8.9	446.1
S6	62	Female	8.3	12.7	634.7
S8	69	Male	7.5	3.9	197.2
S16	57	Female	7.9	5.4	270.9
S20	48	Male	7.7	11.7	583.2
S23	64	Female	7.9	16.7	837.1
S28	59	Male	8.8	5.8	291.4

^†^RNA Integrity Number (RIN) was calculated using Agilent 2100 expert software.

### RNA-Seq library preparation and sequencing

The RNA-Seq libraries for next-generation sequencing (NGS) were generated with a SMARTer Stranded Total RNA-Seq Kit v2 - Pico Input Mammalian (Takara Bio Inc., Shiga, Japan), according to the manufacturer’s instruction. The stored total RNA samples were thawed and their total RNA concentrations were determined with a NanoDrop ND-1000 (Thermo Fisher Scientific, Waltham, MA) and/or NanoPhotometer NP-80 (Implen GmbH, Munich, Germany) instrument. The cDNA derived from rRNA was depleted by ZapR v2 & R-Probe v2 contained in SMARTer Kit during the procedure. The quality and quantity of each RNA-Seq library was confirmed by the following three different methods: i) use of a high sensitivity DNA kit using an Agilent 2100 Bioanalyzer, ii) quantitative PCR (QPCR) with a Stratagene Mx3005P real-time QPCR system (Agilent Technologies) and the SYBR FAST ROX Low qPCR Master Mix of the KAPA Library Quantification Kit Illumina Platform (Roche Sequencing Solutions Inc., Pleasanton, CA), and iii) measurement with a Qubit 2.0 Fluorometer (Thermo Fisher). The generated libraries were then subjected to cluster generation in a flow cell using a TruSeq PE Cluster Kit v3 (Illumina Inc., San Diego, CA) and sequenced by using a paired-end 100-bp read protocol on a HiScanSQ System (Illumina) with a TruSeq SBS Kit v3 (Illumina). The sequencing and the subsequent data processing were carried out at the NGS Core Facility of the Kyoto Prefectural University of Medicine.

### RNA-Seq data analyses

As for the NGS data QC and mapping processes, base calling was performed by bcl2fastq version 2.20 (Illumina). Generated fastq files were applied to the FastQC version 0.11.9. As the fastp 0.20.1 program simultaneously performs both the QC analysis for input RNA-Seq data and QC filtering to remove the low quality/too short sequences, we used the fastq files as the QC filtered reads generated by fastp with default parameter setting. After the QC, adaptor sequences of illumina TruSeq contained in the remained reads were trimmed by Trimmomatic-0.39 program, and configured the following options referring to the web site: ILLUMINACLIP: TruSeq. 3-PE-2.fa:2:30:10:2:keepBothReads LEADING:3 TRAILING:3 MINLEN:36.

The reads that passed these filters were aligned to the human reference genome sequence (GRCh38, for details see the Usage Notes section) by using STAR version 2.7.3a program. The alignment process by STAR was performed with the options of ‘–runMode alignReads’, ‘–outSAMtype BAM SortedByCoordinate’, and ‘–quantMode TranscriptomeSAM’. The gene expression analyses were performed by ‘rsem-calculate-expression’ program from RSEM version 1.3.3 after generated indexes for reference genome and GTF files by ‘rsem-prepare-reference’.

All of the following statistical analyses were conducted by using R program version 3.6.3. Principal component analysis (PCA) and regression analysis were performed by using ‘prcomp’ and ‘cor’ function in the default packages of R, respectively. Transcripts Per Million (TPM) data from RSEM results was extracted for 7,887 genes with TPM ≥ 1.0 in all seven samples from all assessed 60,164 genes and transformed to common logarithm values for these tests. The correlation among seven samples was tested by the Spearman’s rank correlation coefficient test, and the resulting correlation matrix was drawn with the ‘ggcorrplot’ library of R. In the heatmap analysis, the values were normalized with ‘zFPKM’ and heatmap analysis was performed with ‘pheatmap’ (both the libraries were obtained from Bioconductor). Marker genes reported to show specific expression in different corneal tissues, such as the epithelium, stroma, and endothelium, were selected as follows: *PAX6* and *WNT7* were referred from a representative study that performed a functional analysis of the corneal epithelium^31^; *ALDH3A1*, *CHST6*, *KERA*, and *PTGDS* were extracted from expression markers of the corneal stroma or keratocytes commonly reported in four articles^32–35^; and *ATP1A1*, *TJP1*, *COL8A1*, and *SLC4A11* were selected from highly ranked investigated genes related to the corneal endothelium in a comprehensive review article^36^. The expression data of each gene used in the heatmap were evaluated for the existence of outlier samples by the Smirnov-Grubbs test with the ‘outliers’ library of R.

## Data Records

All raw fastq files produced by RNA-Seq were deposited in the DNA Data Bank of Japan (DDBJ) Sequence Read Archive (DRA)^37^. The expression data set of genes obtained by STAR and RSEM was deposited in the DDBJ Genomic Expression Archive (GEA) with Experiment Accession ID E-GEAD-399. The E-GEAD-399 files contain the information of the Ensembl gene ID and the TPM value of each gene derived from all the samples.

## Technical Validation

### Quality assessment of total RNA and RNA-Seq data

**Fig. 1:**
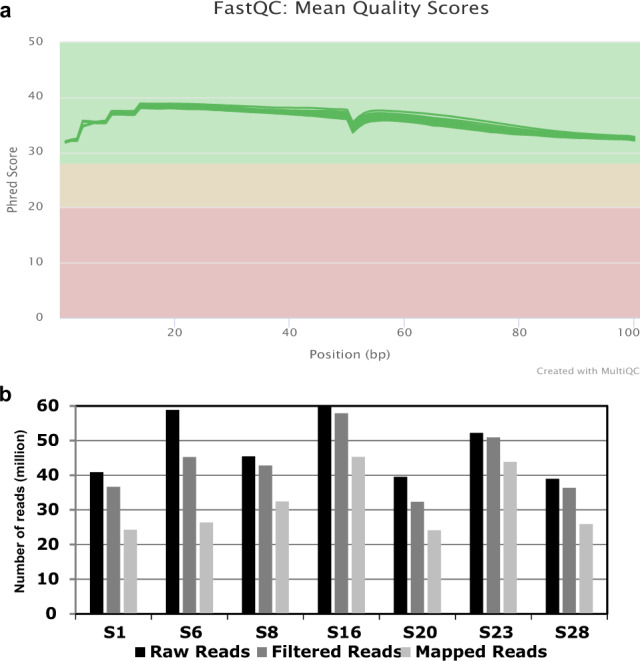
Quality control (QC) results of RNA-Seq data. (**a**) The distribution of the Phred quality score per base sequence based on FastQC for each of the seven samples (green line) generated by multiQC. The original FastQC plot of each sample is shown as Supplementary Figure 3. The different colors of plot area indicate the ranges of Phred quality score as red (<20), orange (20–28), and green (28<). All the post-QC reads were distributed on the green area showing sufficient quality. (**b**) Number of fastq reads (1) without filtering (black), (2) surpassing the QC filters (dark gray), and (3) successful in mapping (light gray).

We obtained sufficient yield (>10 ng) and a RIN value (≥7.0) satisfying the requirements for library preparation (Table 1 and Supplementary Figure 1). The RNA-Seq yielded a number of raw reads derived from seven samples that fell within the range between 38.97 and 59.89 M reads. Regression analysis showed no significant correlation of RNA yield (Supplementary Figure 2a), RIN (Supplementary Figure 2b), or storage period (Supplementary Figure 2c) to the number of raw reads, suggesting that the quality of the produced reads was not affected by the starting RNA and/or the storage condition. All the paired-end reads showed sufficient quality scores after the QC processes (Fig. 1a and Supplementary Figure 3). The filtered reads were mapped to the reference genome within a range between 24.12 and 45.29 M read, relatively less amount of the reads than those of the typical standard read depth for RNA-Seq (~50 M reads/sample), which might be due to the condition(s) of total RNA obtained from the preserved tissues in the eye bank and/or RNA fragmentation resulted in the small library size appeared in some samples (Supplementary Table 2). Overall, the variations in the mapped reads were reduced compared to the raw reads, suggesting that the homogeneity of the RNA-Seq reads among the samples had been improved through the QC processes (Fig. 1b).

**Fig. 2:**
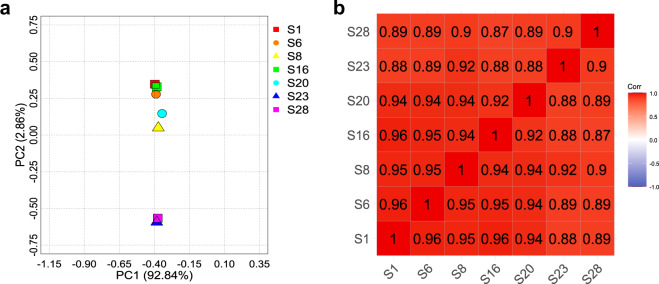
Homogeneity of RNA-Seq data among the samples. PCA (**a**) and the analysis of correlation (**b**) among the RNA-Seq data were performed by TPM values from the selected genes. (**a**) X- and Y-axis shows the principal component 1 (PC1) and PC2 with each contribution rate, respectively. (**b**) The values shown within the correlation matrix indicate the Spearman’s rank correlation coefficient.

We assessed the homogeneity among the samples by analyzing the correlation of the TPM values of the genes filtered from the RNA-Seq dataset (Supplementary Figure 4 and Supplementary Table 2). The result of PCA showed that all samples were distributed within the narrow range of the first component, where the contribution (92.84%) indicated much higher than that of the second component (2.86%) (Fig. 2a). In addition, the correlation coefficients distributed from 0.87 to 0.96 (Fig. 2b). These results suggested a high correlation of the gene expression pattern among the samples.

### Expression analysis of tissue-specific genes

**Fig. 3:**
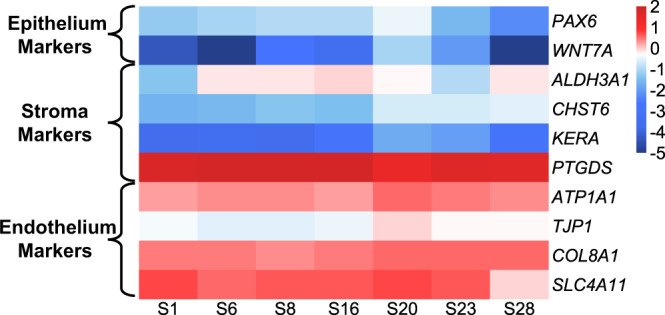
Expression profile of marker genes selected from each layer of corneal tissue. The heatmap indicates the normalized TPM values for each gene and sample. The expression levels of *PAX6* and *WNT7A* were low in the corneal endothelium. The expression levels of *ALDH3A1*, *CHST6*, and *KERA* were low, while the *PTGDS* was highly expressed in the endothelium. The expression levels were high for the genes commonly used as endothelial markers.

We evaluated the expression profile of our current RNA-Seq dataset using genes reportedly expressed in each layer of the cornea (i.e., the corneal epithelium, stroma, and endothelium) (Fig. 3). *PAX6* and *WNT7A*, which are expressed in the corneal epithelium^31^, showed low expression in the endothelium. Most genes with known expression in the stroma^32–35^ showed low expression levels in the endothelium, although the Prostaglandin D2 synthase (*PTGDS*) gene was highly expressed. However, this high expression of *PTGDS* is consistent with a report indicating that *PTGDS* is expressed in both the stroma and the endothelium^38^. By contrast, expression of genes often used as endothelial markers^36^ was generally high. The expression of Tight Junction Protein 1 (*TJP1*, also known as *ZO-1*) was relatively low when compared with other endothelial markers, but this lower expression level was consistent with a previous description of lower expression of *TJP1* mRNA compared to TJP1 protein^26^. The Smirnov-Grubbs test confirmed the absence of outlier samples based on the evaluation of the TPM values from each gene used in the heatmap.

Taken together, the expression data of this corneal endothelium RNA-Seq dataset is considered to be reliable. It should be useful as a reference database of healthy corneal endothelium tissue for the various expression analyses, as well as for future transcriptome analyses including the expression data of non-coding RNAs.

## Usage Notes

The human reference genome sequence (GRCh38) used in STAR alignment process was obtained from Ensembl (ftp.ensembl.org/pub/release-100/fasta/homo_sapiens/dna/Homo_sapiens.GRCh38.dna.primary_assembly.fa). Before aligned, the reference genome was indexed by STAR with ‘--sjdbGTFfile’ option for GTF gene annotation file provided in the same release (http://ftp.ensembl.org/pub/release-100/gtf/homo_sapiens/Homo_sapiens.GRCh38.100.gtf.gz). Note that this GTF file contains the annotation of 60,683 genes, and 60,164 genes were applied to analyses in this study after removing the annotations of tRNA and rRNA referred to ‘RepeatMasker’ track from UCSC Genome Browser (https://genome-asia.ucsc.edu/) on Human Dec. 2013 (GRCh38/hg38).

## Supplementary information

Supplementary Information

## Data Availability

As described in the Methods section, all of the analyses in this study were performed with the following open-access programs: QC checking for RNA-Seq data was performed by FastQC version 0.11.9. (https://www.bioinformatics.babraham.ac.uk/projects/fastqc/). QC results were summarized by multiQC version 1.9. (https://multiqc.info/). QC filtering was performed by using fastp 0.20.1 program with default setting. (https://github.com/OpenGene/fastp). After QC, adaptor sequences were trimmed by Trimmomatic-0.39 program. (http://www.usadellab.org/cms/?page = trimmomatic). All reads were aligned to the human reference genome sequence by STAR version 2.7.3a program. (https://github.com/alexdobin/STAR). Gene expression analyses were performed by RSEM version 1.3.3. (https://deweylab.github.io/RSEM/).
