# Supplementary material for: Transcriptome dataset of human corneal endothelium based on ribosomal RNA-depleted RNA-Seq data
Source: Sci Data. 2020 Nov 20;7:407. doi: 10.1038/s41597-020-00754-1 (PMC7680133; doi:10.1038/s41597-020-00754-1)
Supplement: Supplementary file 1 — Supplementary Information [file 41597_2020_754_MOESM1_ESM.docx]

**Supplementary Information**

**Transcriptome dataset of human corneal endothelium based on ribosomal RNA-depleted RNA-Seq data**

**Yuichi Tokuda^1*^, Naoki Okumura^2*^, Yuya Komori^2^, Naoya Hanada^2^, Kei Tashiro^1^, Noriko Koizumi^2^ & Masakazu Nakano^1^**

^1^Department of Genomic Medical Sciences, Kyoto Prefectural University of Medicine, Kyoto 602-8566, Japan.

^2^Department of Biomedical Engineering, Faculty of Life and Medical Sciences, Doshisha University, Kyotanabe 610-0321, Japan.

^*^These authors contributed equally to this work. Correspondence and requests for materials should be addressed to M.N. (email: manakano@koto.kpu-m.ac.jp)

**Table of contents**

- **Supplementary Figure 1 QC Results of total RNA. ··· P.2**
- **Supplementary Figure 2 Correlation between FASTQ reads and**

**the RNA profiles. ··· P.3**

- **Supplementary Figure 3 Distribution of Phred quality score per base**

**sequence based on FastQC for each sample. ··· P.4**

- **Supplementary Figure 4 Distribution of TPM values among the**

**RNA-Seq data. ··· P.5**

- **Supplementary Table 1 Published RNA-Seq data derived from**

**human corneal endothelium ··· P.6**

- **Supplementary Table 2 Length of library peak, median TPM value,**

**and average read length of each sample. ··· P.7**


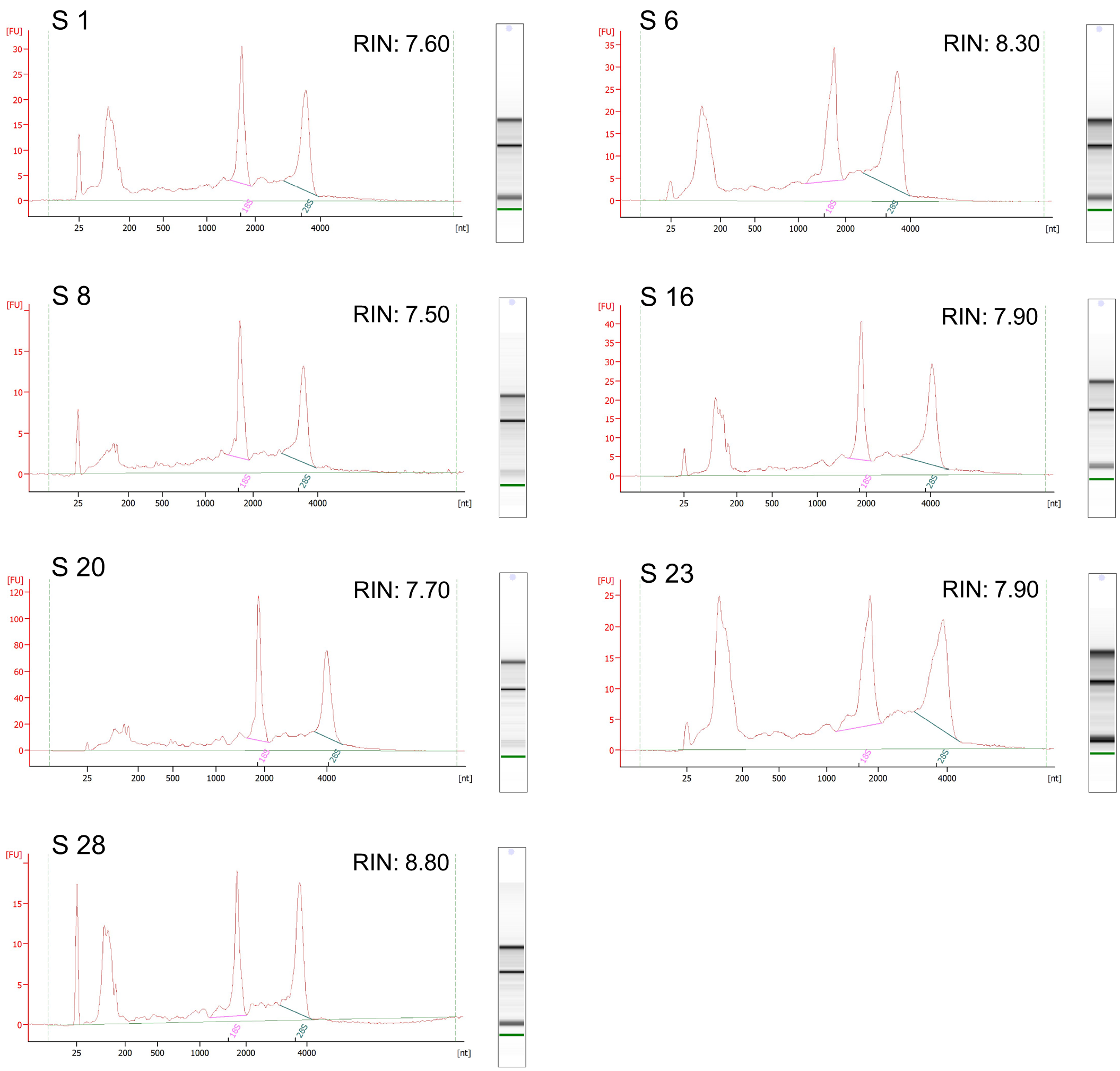


**Supplementary Figure 1. QC Results of total RNA.**

The quality control (QC) analyses of total RNA samples derived from each corneal endothelium tissue were performed with an Agilent 2100 Bioanalyzer and the RNA 6000 Pico Kit (Agilent Technologies). The electropherograms and gel images for each sample were represented by Agilent 2100 Expert Software (Agilent Technologies). Based on the values of RNA Integrity Number (RIN), all the total RNA samples satisfied the required quality (≥7.0) for the NGS library preparation.


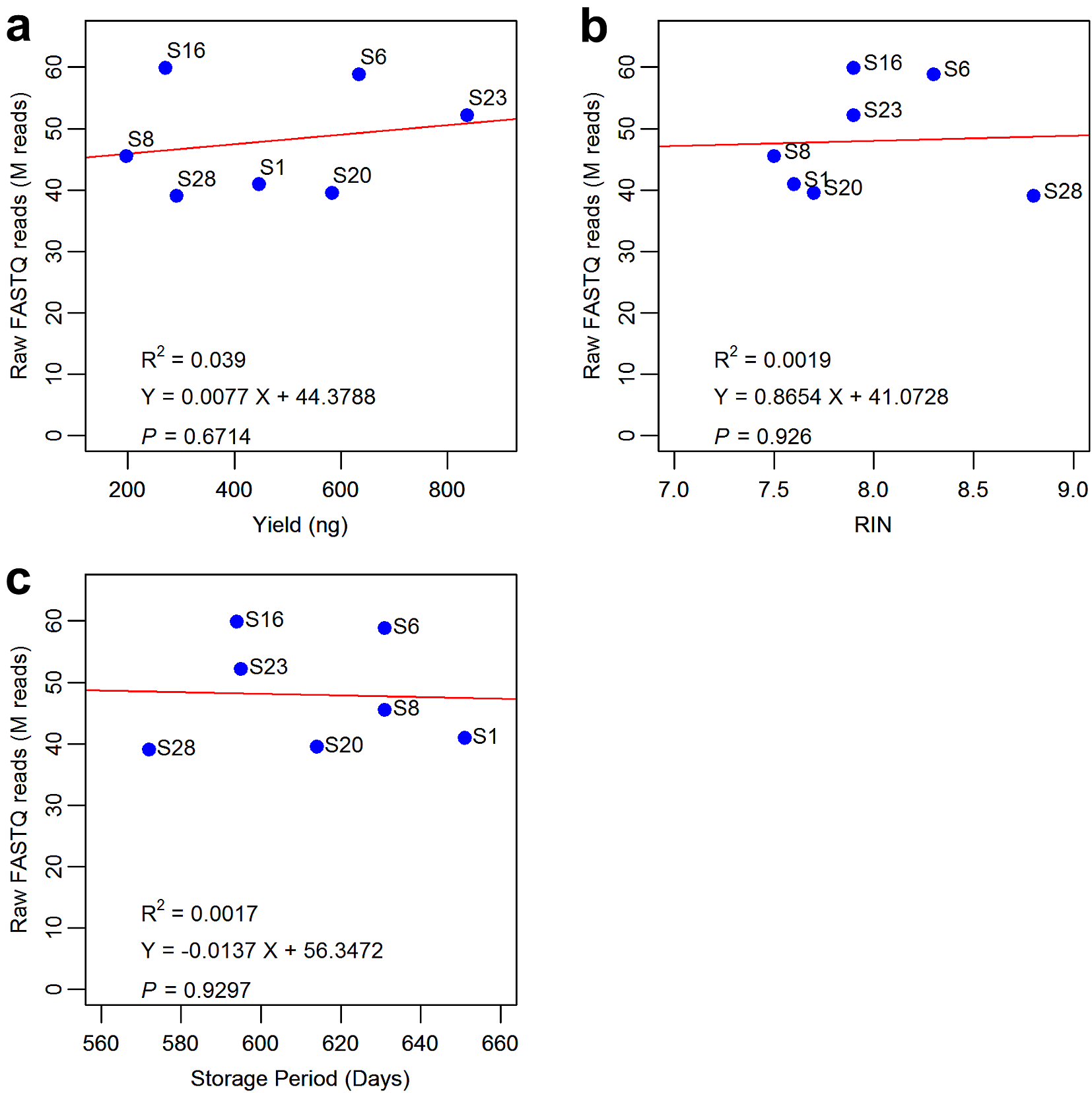


**Supplementary Figure 2. Correlation between FASTQ reads and the RNA profiles.**

The influence of RNA yield (a), RIN (b), or storage period (c) on the produced raw FASTQ reads was assessed by the regression analysis. The regression formula, regression line (red), r-squared value, and *P*-value for the model were calculated with the ‘lm’ (linear regression analysis) function of the R program. No significant correlation was found in any analysis, suggesting that the quality of the produced reads was not affected by the starting RNA and/or the storage condition.


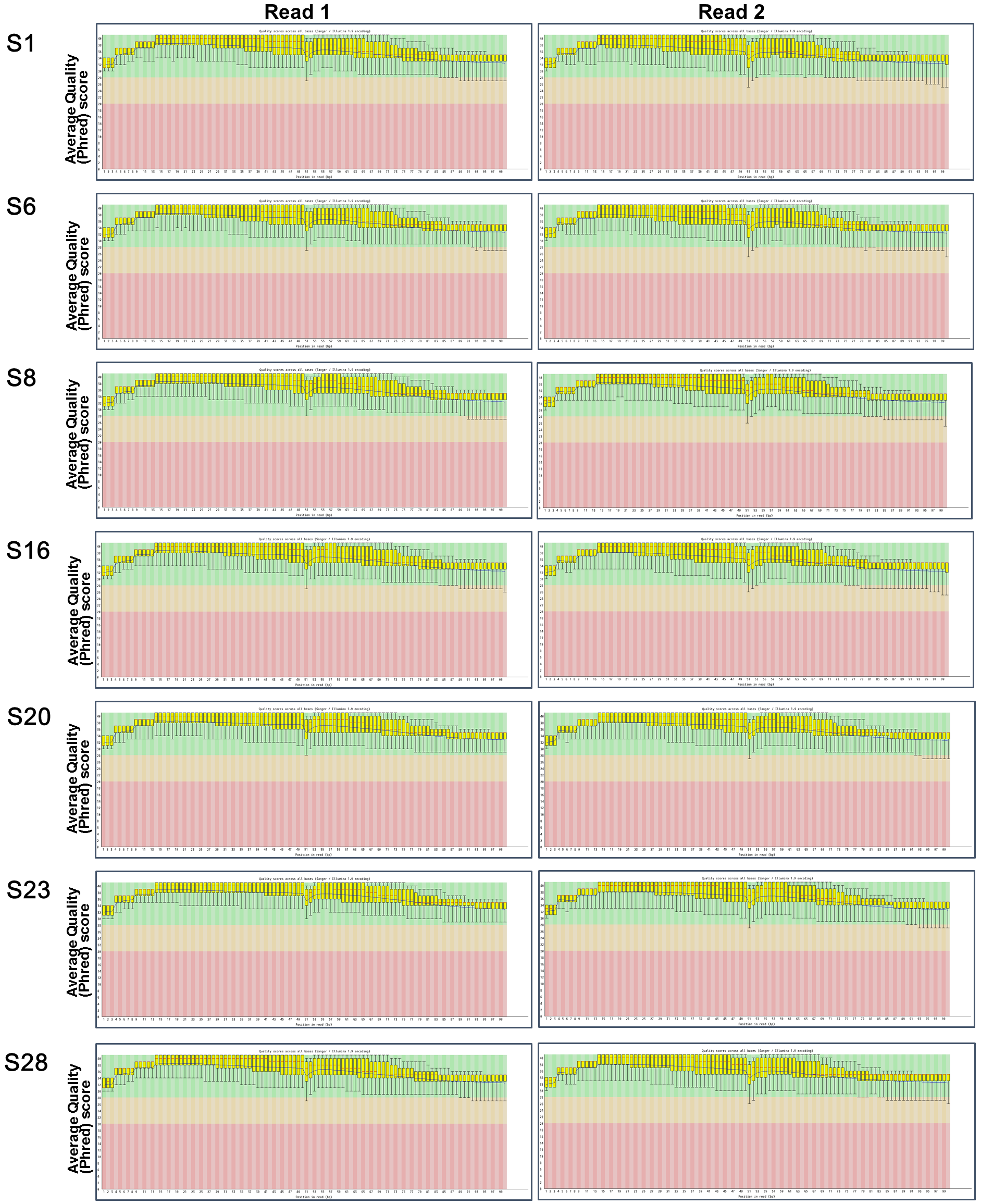


**Supplementary Figure 3**. **Distribution of Phred quality score per base sequence based on FastQC for each sample.**

Red and blue lines indicate median and mean values, respectively. The yellow box indicates the interquartile range (25–75%), with the lower and upper whiskers representing the points of 10% and 90%, respectively.


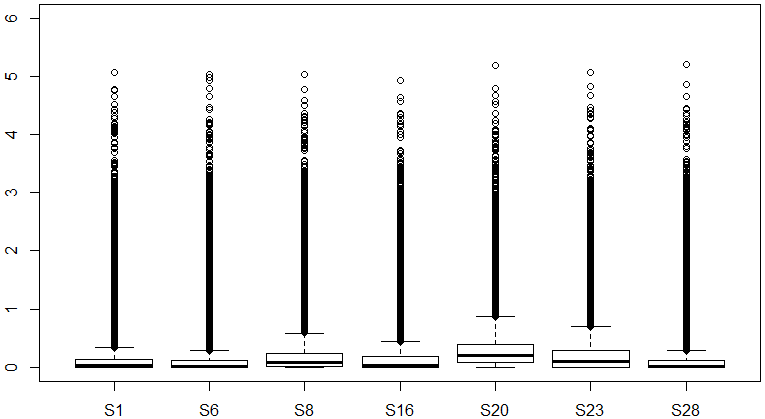


**log_10_ (TPM+1)**

**Supplementary Figure 4. Distribution of TPM values among the RNA-Seq data.**

After calculating whole TPM values from the data of raw FASTQ reads, the distribution of TPM values among the RNA-Seq data was represented as box-whisker plots in common logarithm. Median TPM values of each RNA-Seq data are also summarized in Supplementary Table 2. Based on these data, the genes that showed TPM values ≥ 1.0 were extracted (7,887 genes) from all 60,164 genes included in the RNA-Seq dataset, and then used for PCA and Spearman's rank correlation test shown in Figure 2. The results suggested that the gene expression pattern is highly correlated among the samples.

**Supplementary Table 1. Published RNA-Seq data derived from human corneal endothelium.**

| PMID  [Ref^†^] | Authors | Title | Journal | GEO Accession  Number | Sequencing Kit and Platform | RNA Type | Material |
| --- | --- | --- | --- | --- | --- | --- | --- |
| 23257286  [25] | Chen, Y.  et al. | Identification of novel molecular markers through transcriptomic analysis in human fetal and adult corneal endothelial cells. | *Human Molecular Genetics*  **22**, 1271-1279 (2013) | GSE41616 | Library kit : TruSeq (Illumina)  Sequencer : HiSeq 2000 (Illumina)  Read: Single-end 50-bp | mRNA | Sample: 3 normal adult samples and 2 fetal ocular samples  Source: Eye bank  Tissue: Corneal endothelial layer |
| 26337789  [26] | Frausto, R. F.,  Le, D. J.  & Aldave, A. J. | Transcriptomic Analysis of Cultured Corneal Endothelial Cells as a Validation for Their Use in Cell Replacement Therapy. | *Cell Transplantation*  **25**, 1159-1176 (2016) | GSE65991 | Library kit: PrepX Complete ILM DNA Library Kit (WaferGen Biosystems)  Sequencer : HiSeq 2500 (Illumina)  Read: Single-end 50-bp | mRNA | 1. Sample: 3 adult donors  Source: Eye bank  Tissue: Descemet membrane with endothelial cells, *ex vivo*/primary cultured  2. Sample: 3 adult donors  Source: Eye bank  Tissue: Descemet membrane with endothelial cells |
| 28654985  [27] | Chung, D. D. et al. | Transcriptomic Profiling of Posterior Polymorphous Corneal Dystrophy. | *Investigative Ophthalmology & Visual Science*  **58**, 3202-3214 (2017) | GSE90489 | Library kit: KAPA Stranded mRNA-Seq Kit (KAPA Biosystems)  Sequencer : HiSeq 3000 (Illumina)  Read: Single-end 50-bp | mRNA | Sample: 2 patients with posterior polymorphous corneal dystrophy and 2 age-matched donors  Source: Eye bank  Tissue: Descemet membrane with corneal endothelium cells, *ex vivo*/primary cultured |
| 31233731  [28] | Chung, D. D. et al. | Alterations in GRHL2-OVOL2-ZEB1 axis and aberrant activation of Wnt signaling lead to altered gene transcription in posterior polymorphous corneal dystrophy. | *Experimental Eye Research*  **188**, 107696 (2019) | GSE126487 | Library kit: KAPA Stranded mRNA-Seq  Kit (KAPA Biosystems)  Sequencer: HiSeq 3000 (Illumina)  Read: Single-end 50-bp | mRNA | Sample: 1 patient with posterior polymorphous corneal dystrophy and 1 age-matched donor  Source: Eye bank  Tissue: Descemet membrane with corneal endothelium cells |
| 32366916  [29] | Frausto, R. F. et al. | Phenotypic and functional characterization of corneal endothelial cells during in vitro expansion. | *Scientific Reports*  **10**, 7402 (2020) | GSE121922  GSE132204 | Library kit: KAPA mRNA HyperPrep Kit (KAPA Biosystems).  Sequencer: HiSeq 3000 (Illumina)  Read: Single-end 50-bp | mRNA | Sample: 2 donors  Source: Eye bank  Tissue: Endothelial cells from Descemet membrane, primary cultured |

^†^The numbers correspond to those of the references in the Reference section of the main text.

**Supplementary Table 2. Length of library peak, median TPM value, and average read length of each sample.**

| Sample | Library Peak (bp)^†^ | Median TPM^‡^ | Average Input Read Length (bp)^§^ |
| --- | --- | --- | --- |
| S1 | 199 | 0.15 | 164 |
| S6 | 173 | 0.15 | 145 |
| S8 | 227 | 0.34 | 176 |
| S16 | 295 | 0.20 | 189 |
| S20 | 227 | 0.77 | 147 |
| S23 | 332 | 0.47 | 194 |
| S28 | 274 | 0.20 | 172 |

^†^Calculated by Agilent 2100 Expert Software based on the results of Agilent Bioanalyzer High Sensitivity DNA kit.

^‡^TPM values were calculated by ‘rsem-calculate-expression’ of RSEM program. Medians were calculated after excluding the values that showed 0.0.

^§^Calculated by STAR program based on the alignment results.
